# New standards in HER2-low testing: the CASI-01 comparative methods study

**DOI:** 10.1016/j.ebiom.2025.105919

**Published:** 2025-09-12

**Authors:** David J. Dabbs, Emina Torlakovic, Søren Nielsen, Suzanne C. Parry, Jing Yu, Catherine Stoos, Beth Clark, Henrik Høeg, Jeppe Thagaard, Seshi R. Sompuram, Stephen P. Naber, Yukako Yagi, James Sayre, Kodela Vani, Mélissande Cossutta, Francoise Soussaline, Alexandre Papine, Nils A. t'Hart, Matthias J. Szabolcs, Bharat Jasani, Mary Kinloch, Luis Chiriboga, Keith Miller, Steve Bogen

**Affiliations:** a CASI Steering Committee Member, USA; b Department of Pathology, University of Pittsburgh Medical Center, Pittsburgh, PA, USA; c Department of Pathology and Laboratory Medicine, University of Saskatchewan & Saskatoon Health Authority, Royal University Hospital, Saskatoon, Saskatchewan, Canada; d NordiQC, Department of Pathology, Aalborg University Hospital, Aalborg, Denmark; e Department of Immunocytochemistry and In Situ Hybridisation, UKNEQAS-ICC/ ISH, London, United Kingdom; f Department of Pathology, Nebraska Methodist Health System, Omaha, NE, USA; g Visiopharm A/S, Hørsholm, Denmark; h Boston Cell Standards Inc., Boston, MA, USA; i Department of Pathology & Laboratory Medicine, Tufts Medical Center, Boston, MA, USA; j Department of Pathology, Memorial Sloan Kettering Cancer Center, New York, NY, USA; k Department of Biostatistics, University of California (UCLA), Los Angeles, CA, USA; l Imstar Dx, Paris, France; m Department of Pathology, Isala, Zwolle, the Netherlands; n Department of Pathology & Laboratory Medicine, New York-Presbyterian/Columbia University Irving Medical Center, New York, NY, USA; o Division of Cancer and Genetics, School of Medicine, Cardiff University, Heath Park, Cardiff, CF14 4XN, United Kingdom; p Department of Pathology & Center for Biospecimen Research and Development, NYU Grossman School of Medicine, New York, NY, USA; q Department of Research Pathology, The Cancer Institute, University College London, United Kingdom

**Keywords:** HER2, Immunohistochemistry, Calibration, Analytic sensitivity, Dynamic range

## Abstract

**Background:**

The introduction of Trastuzumab deruxtecan (T-Dxd) has exposed clinically significant limitations in accurately detecting HER2-low expression testing when using immunohistochemistry (IHC) assays originally developed to detect HER2 over-expression. While HER2 testing is widely used to determine T-Dxd eligibility, no HER2-low assay was ever validated against HER2 protein expression.

**Methods:**

To address this pressing need, the Consortium for Analytic Standardization in Immunohistochemistry (CASI) conducted the CASI-01 study, involving 54 IHC laboratories across Europe and the U.S. The study aimed to identify optimal assay conditions for accurate HER2 testing, differentiating between HER2 overexpression (3+) for Trastuzumab eligibility and HER2-low expression (1+ or ultra-low) for T-Dxd eligibility. The conventional FDA-cleared HER2 assay (“predicate”) was compared with higher-sensitivity assays using pathologist versus image analysis readouts. HER2 overexpression was validated against HER2 gene amplification via *in situ* hybridisation (ISH), while HER2-low accuracy was evaluated using newly introduced HER2 reference standards and a novel IHC parameter–dynamic range.

**Findings:**

CASI-01 revealed variability in predicate HER2 assays, with detection thresholds ranging from 30,000 to 60,000 among laboratories. Despite this variability, these assays demonstrated high accuracy for identifying HER2 overexpression (3+), with 85.7% (18/21) sensitivity (95% confidence limits 63.66–96.95%) and 100% (49/49) specificity (95% confidence limits 92.75–100%), though sensitivity may have been limited by the use of older tissue specimens, with loss or reduced expression levels of the HER2 protein. However, these same assays exhibited poor dynamic range for detecting HER2-low scores. Enhanced analytic sensitivity of IHC assays combined with image analysis overcame this limitation with HER2-low scores, achieving a six-fold improvement (p = 0.0017).

**Interpretation:**

IHC assays with detection thresholds in the range of 30,000–60,000 HER2 molecules per cell yield accurate results for determination of Trastuzumab eligibility (HER2 3+) but fail to demonstrate the dynamic range for accurate HER2-low scores. Enhanced analytic sensitivity of HER2 assays combined with image analysis addresses this critical gap in HER2-low testing. More generally, CASI-01 introduces pivotal advancements in precision medicine: (a) the importance of reporting IHC analytic sensitivity and ability to demonstrate an assay dynamic range, and (b) image analysis can surpass pathologist readout accuracy in specific clinical contexts.

**Funding:**

This work was supported by the 10.13039/100000054National Cancer Institute of the 10.13039/100000002National Institutes of Health under Award Number R44CA268484.

**Research in context**

**Evidence before this study**

This study addresses whether and how HER2 IHC assays used in clinical trials to determine patient eligibility for Trastuzumab and Trastuzumab deruxtecan can be reliably reproduced in clinical practice. A comprehensive PubMed review of literature on inter-laboratory discrepancies in clinical immunohistochemistry (IHC) testing (title/abstract search terms “immunohistochemistry” coupled with “inter-laboratory”, or “discrepancies”, or “survey”, or “HER2”, for the time period 1/1/2010–12/31/2024), revealed significant analytic and interpretive variability across IHC laboratories. Recent studies have explored the application of reference materials for measuring inter-laboratory assay differences, but none have employed them to define acceptable IHC assay performance or to validate HER2 assays.

Additionally, three co-authors of this study (ET, SZP, SN) oversee three different national or international IHC proficiency testing (PT) organisations (CBQA, UKNEQAS-ICC/ISH, NordiQC) that routinely report such inter-laboratory differences that are observed in PT surveys. A review of regulations governing IHC laboratory accreditation and assay validation requirements (CLIA, FDA, CLSI) confirmed that, unlike blood testing, IHC lacks reference standards, calibration, measurement of analytic sensitivity, or statistical control procedures.

**Added value of this study**

CASI-01 demonstrated the use and transformative impact of IHC reference materials in validating clinical HER2 testing or any other biomarker. The study breaks new ground by applying newly developed IHC HER2 reference materials as an objective accuracy standard. In so doing, the study establishes HER2-low assay dynamic range as a critical new parameter for IHC assay performance evaluation. Previously, the absence of an assay gold standard for HER2-low testing posed significant challenges for translating clinical trial results to clinical practice. CASI-01 identifies poor dynamic range as a key source of HER2-low testing variability and identifies a viable solution. The study demonstrates that the assay dynamic range problem in the HER2-low spectrum can be solved using more sensitive HER2 assays combined with objective image analysis readouts.

**Implications of all the available evidence**

These findings mark a pivotal transition in IHC companion diagnostics, evolving the practice from an unregulated “stain” approach to a robust “assay” model incorporating calibration, reference standards, analytic sensitivity metrics, and statistical process control. CASI-01 provides compelling evidence to support multiple recently published calls for immunohistochemistry regulatory reform, reinforcing the need for systematic adoption of assay standards to improve diagnostic accuracy and patient outcomes.

## Introduction

Recent data from the DESTINY/Breast04 (DB04) and 06 (DB06) clinical trials demonstrated the efficacy of a new antibody-drug conjugate (ADC), Trastuzumab deruxtecan, for treatment of patients with unresectable metastatic breast cancer disease who failed previous treatment regimens.^1^^,^^2^ T-Dxd, a conjugate of a HER2-directed antibody conjugated to deruxtecan, a topoisomerase inhibitor, demonstrated significant benefit in progression-free survival. In the DB04 trial, the eligibility criteria for treatment were HER2 scores of 1+ or 2+ and *in situ* hybridisation (ISH)-negative (unamplified).^2^ The pathologist's readout/scoring and its inter- and intra-observer reproducibility are problematic because accurate identification of low levels of HER2 protein expression are challenging for pathologists.^3^, ^4^, ^5^, ^6^ Additional confounding variables include lab-to-lab differences in analytic sensitivity and fluctuations in IHC assay performance within any single lab. Highlighting these difficulties, the DAISY trial, demonstrated that 30% of patients with HER2 0 expression, who would not otherwise be eligible for treatment, responded to T-Dxd.^7^ Moreover, a retrospective analysis of HER2 0 cases found that approximately 30% were judged as 1+ upon repeat staining.^8^ This finding suggests that the HER2 assay and readout employed in the DB04 and DAISY clinical trials potentially lacked sufficient analytic sensitivity that would allow for accurate and reproducible detection of tumours with low HER2 expression levels. The DB06 trial data further supported this finding, documenting that patients with the slightest degree of HER2 staining, with a new HER2 scoring category termed “ultralow”, also benefited from T-Dxd.^1^ Consequently, pathologists are now tasked with reproducibly and accurately distinguishing the slimmest of expression differences, distinguishing HER2-negative from HER2-low and HER2-ultralow. Moreover, this distinction must be accomplished with HER2 assays that lack reference standards to calibrate analytic sensitivity.

Despite the DB04 trial showing an exciting treatment success for patients, it is important to note that the HER2 assays in widespread use were not developed for detecting low HER2 protein expression levels.^9^ Instead, the assays were designed to detect high HER2 membrane expression (3+ and 2+/ISH+) indicating likelihood of response to a different class of drugs (e.g., Trastuzumab), which work by interfering with HER2 signalling.^10^ Historically, the 1+ score was considered “negative” and therefore received scant attention from pathologists, as it clinically was considered no different than a score of “0”. Accordingly, HER2 proficiency tests from the College of American Pathologists, NordiQC, and UKNEQAS-ICC/ISH reported HER2 0 and 1+ as a single category.

Consequently, there is a need to revisit how laboratories test for HER2 protein expression assessment, especially at the low end of the range. Essentially, the HER2 IHC assays developed for the detection of HER2 overexpression are being repurposed for the detection of low/very low levels of HER2 expression. However, assays developed to detect overexpression of HER2 may be missing tumours that express clinically relevant levels of HER2 in the context of T-Dxd treatment. To explore relevance of analytical sensitivity for diagnostic accuracy covering the spectrum of different levels of HER2 expression, we conducted a study examining the analytic sensitivities of commonly used commercial HER2 IHC assays, with a special emphasis on the assay used for the DB04 trial. This study addresses the effectiveness of the current HER2 IHC assays' analytic sensitivity for the intended purpose of directing patients either for Trastuzumab (over-expression of HER2) or T-Dxd (low HER2 expression).

## Methods

### Overview

The CASI-01 study included three main phases: (1) creation of the survey slides, (2) distribution of the survey slides and staining at participating IHC laboratories, and (3) collecting the slides, quantifying calibrator stain intensity and readout of the stained TMAs and analysing the data.

### Construction and characterisation of a HER2 tissue microarray

**Supplementary Fig. S1:**
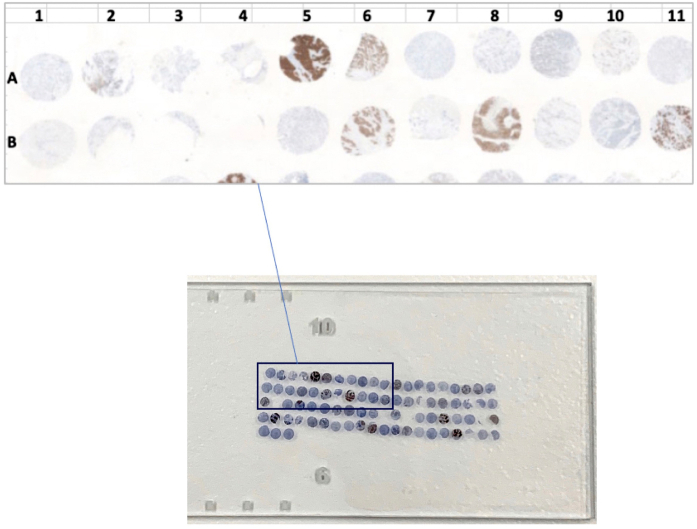


**Supplementary Fig. S2:**
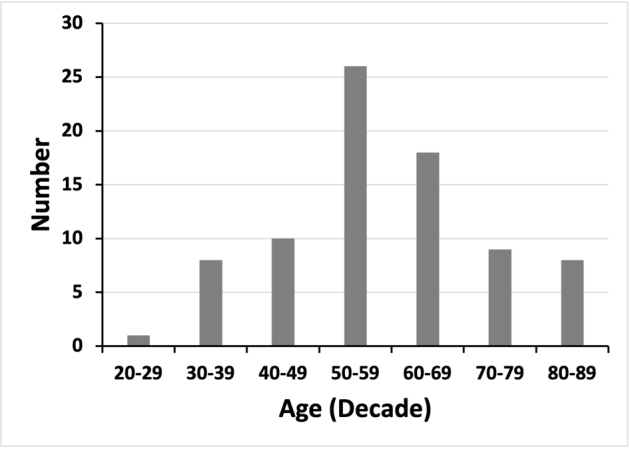


**Supplementary Fig. S3:**
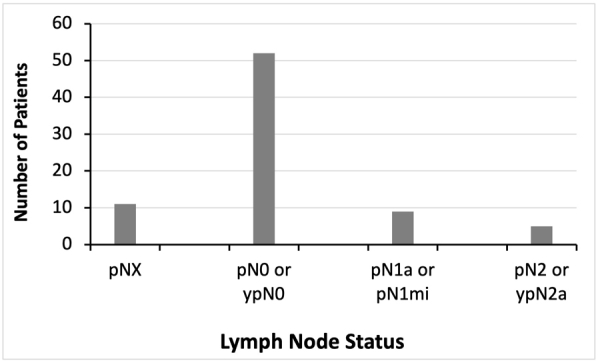


**Supplementary Fig. S4:**
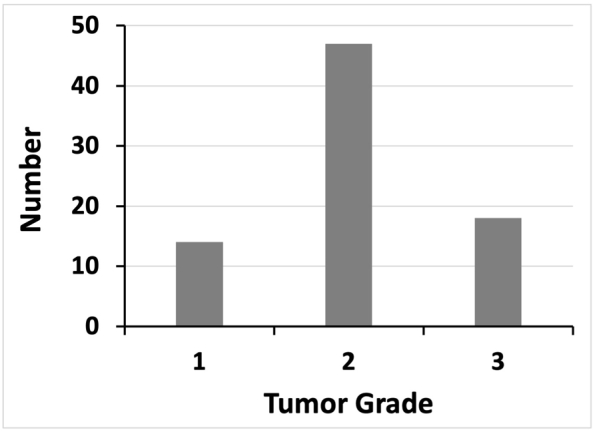


The TMA was prepared from archival breast adenocarcinoma samples with known HER2 status (0–3+) from the Tufts Medical Center Biorepository, under their approved Institutional Review Board protocol for the use of de-identified patient samples in paraffin blocks. Each TMA had 80 cores with equally divided HER2 scores in each category (0–3+). From the paraffin blocks, 1 mm diameter cores were extracted to build an 80-member TMA, in four rows of 20 cores (Supplementary Fig. S1). Three irrelevant orientation cores were also included. Serial 4-micron thick sections were mounted on slides and baked at 50 °C for 1 h prior to shipping to participating laboratories. The age distribution of the patients from whom the tumour cores were derived is shown in Supplementary Fig. S2, lymph node status in Supplementary Fig. S3, and tumour grade in Supplementary Fig. S4. Chromogenic *in situ* hybridisation (ISH) testing of the TMA cores was performed at Memorial Sloan Kettering Cancer Center, Department of Pathology & Laboratory Medicine.

### Ethics approval

De-identified tissue cores for the TMA were procured from archival paraffin blocks through the Tufts Medical Center Tissue Biorepository, Boston, MA under approved protocol #10247 entitled “Retention of De-identified Archival Paraffin Blocks” from the Tufts Medical Center/Tufts University Institutional Review Board (IRB). A Tufts Medical Center breast pathologist and Medical Director of the Tufts Medical Center Tissue Biorepository (SPN) selected suitable cases, captured relevant (de-identified) clinical information, and marked the areas of each paraffin block for use in creating the TMA. Written informed consent was not obtained, as the study utilised fully de-identified, archived human tissue samples provided by a certified biorepository. Per applicable regulations and institutional policies, research involving such materials does not require patient consent.

### HER2 calibrator slides

**Supplementary Fig. S5:**
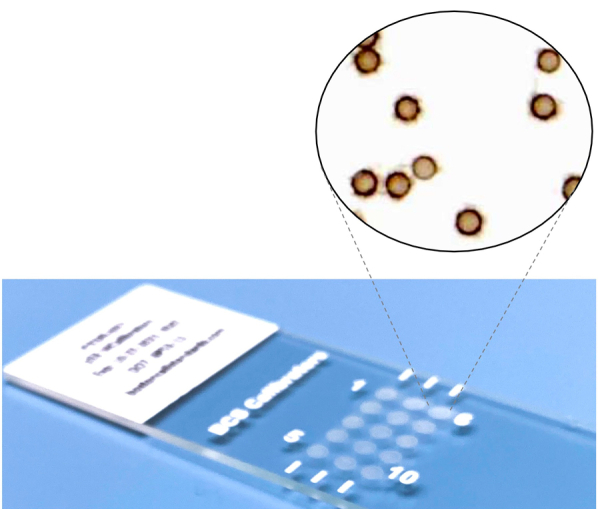


The HER2 calibrators (Boston Cell Standards, Boston MA) are synthetic samples comprised of the HER2 antibody epitope with defined analyte concentrations (26–1400 × 10^3^ HER2 molecules) covalently coupled to cell-sized (8-micron diameter) clear microbeads.^11^ The number of HER2 molecules is in units of equivalent reference fluorophores (ERF), as defined below. Each HER2 peptide has an attached fluorescein positioned away from the epitope, for quantification. Microbeads with each HER2 concentration are pipetted onto microscope slides, ten concentrations in total (Supplementary Fig. S5). The microbeads are spatially distributed so that microbeads with each HER2 concentration are in a separate spot on the slide. In addition, there are five other spots with microbeads bearing an antigenically irrelevant peptide, to serve as a negative control (Supplementary Fig. S5). All peptide-coated microbeads are formalin-fixed in the presence of an antigenically irrelevant protein. Upon staining, the assay detects HER2 on the microbeads and produces a colour proportional to the HER2 concentration. The stained microbeads are then photographed and analysed for stain intensity (see Methods: Image analysis readout of TMA scores).

HER2 concentrations are quantified against National Institute of Standards and Technology (NIST) Standard Reference material (SRM) 1934, a fluorescein standard commonly used in flow cytometry. The unit of measure associated with this SRM are “equivalent reference fluorophores” (ERF).^12^^,^^13^ The ERF value links the microbead fluorescence intensity to a molecular (molar) concentration. NIST SRM 1934 is a soluble fluorescein standard. The ERF unit designation creates a link between the soluble fluorescein standard and the insoluble fluorescein anchored on the microbead surface. Since we are quantifying the HER2 lower limit of detection (LOD) on 8-micron diameter microbeads and comparing it to tumour cells of varying sizes, we use the term “per cell equivalent”. The phrase refers to the number of HER2 molecules per 8-micron diameter microbead, a surrogate for the diverse variety of tumour cells found in a tissue section.

### HER2 IHC survey of laboratories

The slides were shipped to participating IHC laboratories either from the study sponsor (Boston Cell Standards, Boston MA), NordiQC, Aalborg, DK, or UKNEQAS-ICC/ISH, London, UK to a total of 54 laboratories across Europe and the U.S. Each IHC laboratory performed HER2 IHC testing per the laboratory's established protocol for patient testing. Data regarding the specific methods each laboratory used were also collected. All stained slides were eventually returned to the study sponsor for assessment of calibrators and stained TMAs.

### Pathologist readout of TMA scores

Four expert breast pathologists (DJD, JY, CS, BC) divided the slides for HER2 scoring per the ASCO/CAP criteria. This effort required scoring approximately 80 cores per slide × 54 slides, more than 4000 cores. Consequently, each core was judged by only one pathologist. This protocol matches current patient-care practice, whereby most HER2 IHC stains are reviewed and scored by one pathologist. In addition, pathologists made note of a category of “ultralow” HER2, defined as incomplete or faint staining in ≤10% of invasive cancer cells, according to 2023 ESMO guidelines.^14^ In all calculations, both for manual scoring and with image analysis, ultralow scores were tabulated as 0.5.

### Image analysis readout of TMA scores

Two different systems for image analysis were included in the study:

#### Visiopharm digital image analysis

The TMA slides were scanned on a Roche DP-200 slide scanner using a 20× objective lens. To manage the TMA cores, the whole slide images were dearrayed using Visiopharm Tissuearray (Hoersholm, DK). All TMA cores were then analysed with the commercially available Visiopharm HER2 image analysis APP (ID 10185).^15^ The APP detects tissue and segments invasive tumours while excluding normal and *in situ* components. Then, it identifies tumour cells and classifies them based on their membrane staining and completeness. Finally, single-cell distributions are then used to produce a 0–3+ HER2 score for each tissue core according to the latest ASCO/CAP 2023 guidelines.

#### ImstarDx digital image analysis

WSIs corresponding to HER2 IHC TMAs were produced using a fully motorised Olympus BX63 Pathfinder microscope and a Märshäuzer Slide Express slide loader, equipped with a 10× or 20× objective. The WSIs were analysed using the PathoScan platform (Imstar Dx, Paris, FR) including the Tumour Marker HER2 IHC module “TM HER2 IHC” for the automated quantification of HER2 IHC staining.^16^^,^^17^ The TM HER2 IHC computer vision-based module runs in two fully automated steps. The first line of algorithms uses image colourimetric and morphological features to perform a subcellular segmentation of cancer cells by identifying cell nuclei and plasma membranes. The second line of algorithms quantifies the HER2 staining intensity of the pixels identified at the plasma membrane of cancer cells at the first step. The measured HER2 stain intensity (4096 stain intensity channels) was ultimately translated to a 5-level scale of HER2 IHC scores for each tissue core: 0, 0.5+ (ultralow), 1+, 2+, and 3+.

### Quantification of assay lower limit of detection (LOD)

The HER2 calibrators were photographed with a Zeiss Imager.Z2 photomicroscope (Jena, Germany). Immunohistochemical stain intensity was calculated using a custom image analysis algorithm developed by Applied Spectral Imaging, Yokneam, Israel. The method is similar to one that was previously described.^18^ To calculate bead tumour intensity, images were first converted from RGB tumour format to greyscale intensity and then segmented to identify round beads using Hough transform methods for segmentation of circles. Because all images were acquired at a known magnification, parameters were established for an allowable range of bead radii. After image segmentation, additional logic was used to remove segmentation errors, such as where overlapping beads were found, and then quantify stain values for each bead. Stain concentration was estimated as the dot product of the measured RGB values at each point with the known RGB profile of the diaminobenzidine stain.

All measurements were performed in duplicate and averaged. From these quantitative data, the lower limit of detection was calculated as the HER2 concentration that produces a stain intensity equal to that of the negative controls + 3 standard deviations (SD). In other words, the LOD (sometimes also referred to as “limit of blank”) is the stain intensity that has a 99% probability of being different than background staining. Since each participating laboratory stains one calibrator slide, calculations of LOD that incorporate precision parameters were not feasible.

To calculate LOD, the stain intensity data (y axis) were plotted as a function of HER2 concentration (x axis), both of which are linear axes. This produces a sigmoid-shaped analytic response curve. The data points comprising the upward slope were then used to establish a log regression curve in Microsoft Excel. In our experience, logarithmic regressions are a better fit than linear regressions for immunohistochemical analytic response curves. Alternatively, linear regressions on a semi-log graph are essentially equivalent. In calculating the regression line: (a) data points on the baseline, with stain intensities no greater than the negative control, are not included because a log regression does not model the lower sigmoid curve bend, (b) data points on the analytic response curve plateau are also not included, as the sigmoid curve upper bend is also not well modelled with logarithmic regression curve fitting, (c) the regression curve includes at least three data points and preferably five, and (d) LODs below the lowest calibrator concentration can be calculated if the data points defining the upward slope of the curve are represented. The LOD is calculated by solving for x in the regression line, where y is the mean + 3 SD of the negative control stain intensity.

### Assessment of HER2 IHC dynamic range

The dynamic range of a HER2 IHC assay refers to the span of HER2 expression levels in tumours that produce corresponding changes in the pathologist's scoring (HER2 0–3+). This concept is grounded in the expectation that the morphologic criteria that a pathologist uses to develop a score reliably reflects actual tumour HER2 protein expression. To our knowledge, this has never been tested at the level of resolution that stratification of patients for T-Dxd treatment calls for. Assessing dynamic range involves testing whether this correlation holds true for a given assay, readout method, and range of biomarker concentrations.

Recent reports^3^, ^4^, ^5^ describing difficulty in reproducibly distinguishing HER2-low from HER2-negative tumours (scores 0, ultralow, or 1+) raise the question of whether these scores genuinely reflect differences in HER2 expression. There is an implicit assumption that HER2 protein levels follow a gradient: 1+ > ultralow > 0, but it remains unclear to what extent the scores represent actual HER2 protein expression differences versus variability in the assay or readout. The former relates to dynamic range, the latter to imprecision.

HER2 protein expression is measured via IHC. Assays with greater analytical sensitivity (i.e., lower limits of detection, or LODs) should detect lower levels of HER2 protein. With such assays, cases originally scored as HER2 0 may shift to ultralow, ultralow cases to 1+, etc., as more HER2 protein is detected. Using an iceberg analogy, current HER2 scoring evaluates what is visible above the “waterline” of detection. When analytic sensitivity increases (i.e., the waterline lowers), more signal becomes visible, and some scores shift upward. This increased signal can appear visually as greater stain intensity, a higher number of stained tumour cells, and more complete circumferential membrane staining. The CASI-01 study enables an evaluation of this principle. By comparing commercial HER2 assays and readout methods, we can assess which combinations show stronger correlations between assay sensitivity (LOD) and pathologist scoring—thereby identifying assays with improved dynamic range.

Measuring dynamic range for serum immunoassays requires samples with a range of defined biomarker concentrations. However, there are no such commercially-available defined tumour samples for a HER2 IHC assay. HER2 expression levels in commercially available cell lines are typically characterised qualitatively—“High”, “Medium”, “Low”, or “Negative”. Quantitative HER2 cell lines were recently published,^19^ but are not commercially available.

**Fig. 1:**
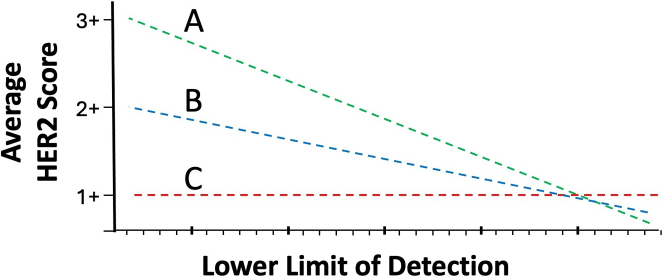
Three hypothetical relationships for the average HER2 score of a TMA (y axis) as a function of analytic sensitivity (LOD). The curves illustrate that HER2 scores may theoretically increase (A, B) or remain unchanged (C) in response to increasing analytic sensitivity (lower limits of detection). A slope of zero (C) would be seen if the assay is unable to measure HER2 expression across the analytic range (x axis). Curves with a higher slope (A) indicate that the assay produces a higher stain intensity for each additional increment in analytic sensitivity as compared to a curve with a lower slope (B).

Even with a HER2 calibration curve, there is no readily available method for quantifying HER2 protein expression in formalin-fixed archival patient samples. Therefore, in assessing the accuracy of HER2 assay at the low end of the measurement scale, we incorporated a modified experimental design. Since it's not yet feasible to determine HER2 assay accuracy for each individual breast cancer sample, we correlated the aggregate average HER2 score (from up to 80 different TMA samples) with LOD. With a diverse collection of up to 80 breast carcinoma samples, HER2 assays that are more sensitive (lower LODs) should produce higher average aggregate scores. Less sensitive HER2 stains (higher LODs) should produce lower average aggregate HER2 scores. For example, HER2 assays with a 20,000 LOD should generate higher average HER2 scores than assays with a 60,000 LOD. This relationship between average HER2 score and LOD (Fig. 1) is a measure of dynamic range, as reflected in the slope.

The concept of assessing the dynamic range by examining the correlation of the aggregate average HER2 score as a function of analytic sensitivity (or measured LOD) is illustrated in Fig. 1, with a hypothetical curve “A”. As the LOD decreases (increasing analytic sensitivity), an increasing number of samples should turn positive in the assay. For example, some HER2 0 samples will become ultralow (positive), ultralow samples may become 1+, etc. As a result, the average HER2 score rises, but only if the score is affected by HER2 expression levels.

Fig. 1, curve C illustrates a hypothetical situation of a HER2 IHC assay that is unresponsive to HER2 expression levels. Regardless of the level of HER2 expression, the scores are unchanged. For curve C, the lack of increased HER2 scores may be due to the fact that the HER2 expression levels are below the assay LOD. Alternatively, it can be due to variability in the assay or readout method, which is so great that it obscures any differences in HER2 expression. Fig. 1 curve B depicts a hypothetical situation between the extremes of A and C. The slope of the regression line is a measure of dynamic range over the HER2 concentrations on the x axis.

### Calculation of diagnostic accuracy

Diagnostic accuracy of each laboratory is calculated by first converting each HER2 score into a designation of true positive (TP), true negative (TN), false positive (FP), or false negative (FN). These are defined as follows:

TP: The HER2 score correctly indicates that treatment is warranted.

TN: The HER2 score correctly indicates that treatment is not warranted.

FP: The HER2 score incorrectly indicates that treatment is warranted when, in fact, it is not.

FN: The HER2 score incorrectly indicates that treatment is not warranted when, in fact, it is.

The conversion of HER2 scores to TP/TN/FP/FN designations depends on what we consider the correct, or “gold standard”, test result against which all others are compared. For HER2 3+, the ISH HER2 assay is used as the gold standard. For lower HER2 scores, there is no analogous gold standard or surrogate assay quantifying low HER2 protein expression levels. Designating a group of labs and pathologist readers as the gold standard is viewed as arbitrary for this purpose. Therefore, an alternative was devised. In this analysis, the gold standard was set as the consensus result of IHC laboratories. The consensus was analysed based on different groups of IHC laboratories, separated by their HER2 assay LOD. Consequently, the consensus HER2 score for various TMA cores might be different for laboratories having different HER2 analytic sensitivities. This analysis enables an examination of the consequences of variability in LOD on diagnostic accuracy among participating laboratories.

The HER2 score consensus for labs that fit the LOD criterion was calculated in Excel, as the average score for each TMA core, rounded to the nearest HER2 score. For example, an average of 1.7 is rounded to a HER2 2+ score. A score of 0.7 is rounded down to 0.5 if a HER2 ultralow designation is included. If the group does not include the ultralow designation, then it would be rounded up to a HER2 1+ score.

**Supplementary Fig. S6:**
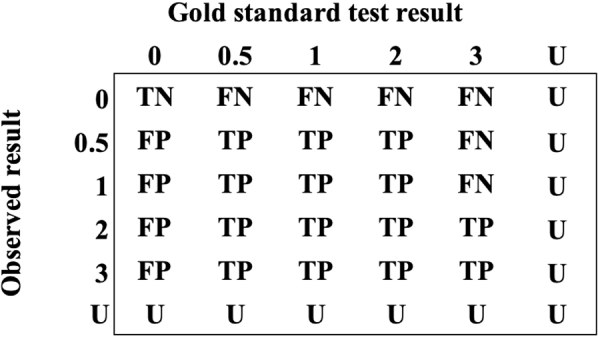


The HER2 score-to-TP/TN/FP/FN conversion (in the context of T-Dxd treatment) was performed based on the logic table displayed in Supplementary Fig. S6 using the INDEX and MATCH lookup functions in Excel. For each result, the logic table (Supplementary Fig. S6) asks whether the patient would have been treated in the same manner as per the gold standard test result. Diagnostic accuracy was then calculated for each laboratory where TP, TN, FP, and FN are the total number of samples per laboratory with that designation (Accuracy = (TN + TP)/(TN + FP + FN + TP)).

### Statistics

Microsoft Excel was used for database management and for generation of the various plots. TMA results were also stored in Excel and transformed to logic tables for evaluating different HER2 assays and readout methods. Diagnostic sensitivity and specificity were also evaluated as a function of analytic sensitivity (LOD) using Excel. IBM SPSS Statistics for Windows, 28.0. Armonk, NY and Stata 15 StataCorp, College Station, TX were used to perform descriptive statistics (means, medians, standard deviations, and percentages) and linear regression analysis. The Clopper–Pearson method was used to calculate 95% confidence intervals for sensitivity, specificity, positive and negative predictive values. Dynamic ranges are evaluated by linear regression slope coefficients. Statistical significance of these coefficients is evaluated by t-tests and expressed with p values, which test for the statistical significance of slope coefficients relative either to one another or relative to a slope of zero. A slope of zero represents an absence of assay dynamic range. The threshold for statistical significance was set at two-sided p ≤ 0.05.

### Role of funders

This work was supported by the National Cancer Institute of the National Institutes of Health under Award Number R44CA268484. The funding agency had no involvement in the design of the study; the collection, analysis, or interpretation of data; the writing of the manuscript; or the decision to submit the manuscript for publication. No individuals received compensation from the funder or any other entity for writing this article.

## Results

### Analytic sensitivity of various commercial assays

**Fig. 2:**
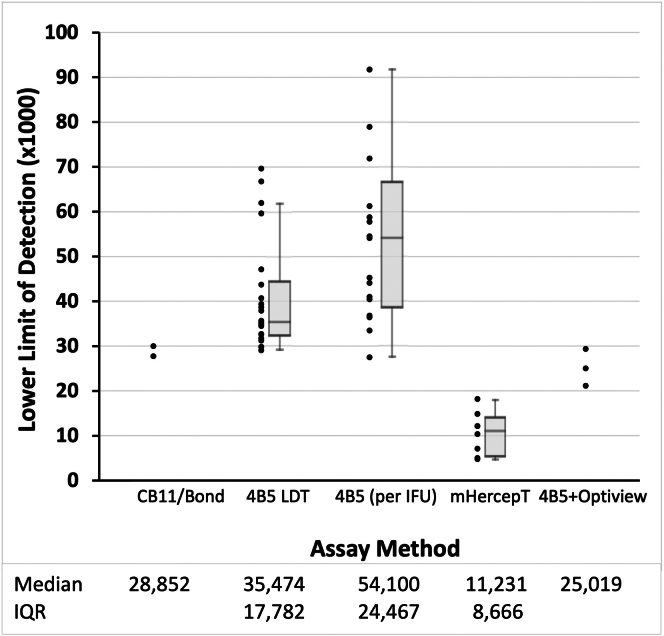
Analytic sensitivity, as measured by the lower limit of detection (y axis), for the various commercial assays used by participating laboratories (x axis). Each dot is a separate laboratory. A box plot is also illustrated with each data set showing the median, 25th and 75th quartiles, calculated exclusive of the median because each group has an even number of samples. Assay nomenclature: “4B5 (per IFU)” is the Roche PATHWAY assay without modification; “4B5 LDT” is the same reagents and instrument but after modification of the protocol, often extending an incubation time; “4B5+Optiview” is the same but coupling the primary antibody to the Optiview detection system; “mHercepT” is the Agilent DG44 monoclonal HercepTest on the Dako Omnis; “CB11/Bond” is the Leica Biosystems CB11primary antibody pre-dilute on a Leica immunostainer. Lower limit of detection (y axis) is in ERF units per cell equivalent (Methods) and is intended to be multiplied ×1000.

Before assessing whether the analytic sensitivities of the various assays are fit for their intended purpose, it's first necessary to measure them. Therefore, we quantified the analytic sensitivity of the various commercial HER2 IHC assays using HER2 calibrators after staining by each participating laboratory. The data for each HER2 assay type are displayed in Fig. 2. Each data point represents a participating laboratory. The results and data generated in the DB04 clinical trial are based on the Ventana IHC HER2 assay “4B5 per IFU”. The DB04 clinical trial assay was identical in its formulation and staining process to the Ventana PATHWAY assay, which is also based on clone 4B5, the Ultraview detection reagent, and per the kit instructions for use (IFU). Therefore, data for “4B5 per IFU” and PATHWAY are treated as equal and methods subsequently named “4B5 PATHWAY”. The majority of labs using that assay have LODs in the 30–60,000 HER2 molecules (ERF) per cell equivalent range.

The “4B5 PATHWAY LDT” group introduced various small protocol adjustments to incubation time and/or epitope retrieval time, rendering it a laboratory developed test (LDT). Fig. 2 data show that the protocol adjustments generally improved inter-laboratory consistency and resulted in more laboratories achieving a higher analytic sensitivity (lower LODs). The data suggest that the lowest LOD this combination of the 4B5 primary antibody, detection reagent, and optimised protocol, can achieve is approximately 30,000 molecules HER2 (ERF) per cell equivalent. This appears to be an analytic sensitivity plateau. This plateau is important in context to a clinical laboratory recommendation in the Discussion section.

When laboratories modified the 4B5-PATHWAY assay by applying the OptiView detection system instead of the recommended UltraView system, the analytical sensitivity was further increased, lowering the LODs to the 20,000–30,000 range (“4B5+Optiview”). Furthermore, the Agilent DG44 monoclonal HercepTest assays displayed even higher analytic sensitivity, with LODs in the 5000–20,000 range. This higher analytic sensitivity of the monoclonal HercepTest been previously reported based the larger number of positive results with patient samples when it was compared to 4B5-PATHWAY assay.^20^^,^^21^ Our study confirms this contention.

### Diagnostic fidelity of HER2 measurement for Trastuzumab eligibility

**Fig. 3:**
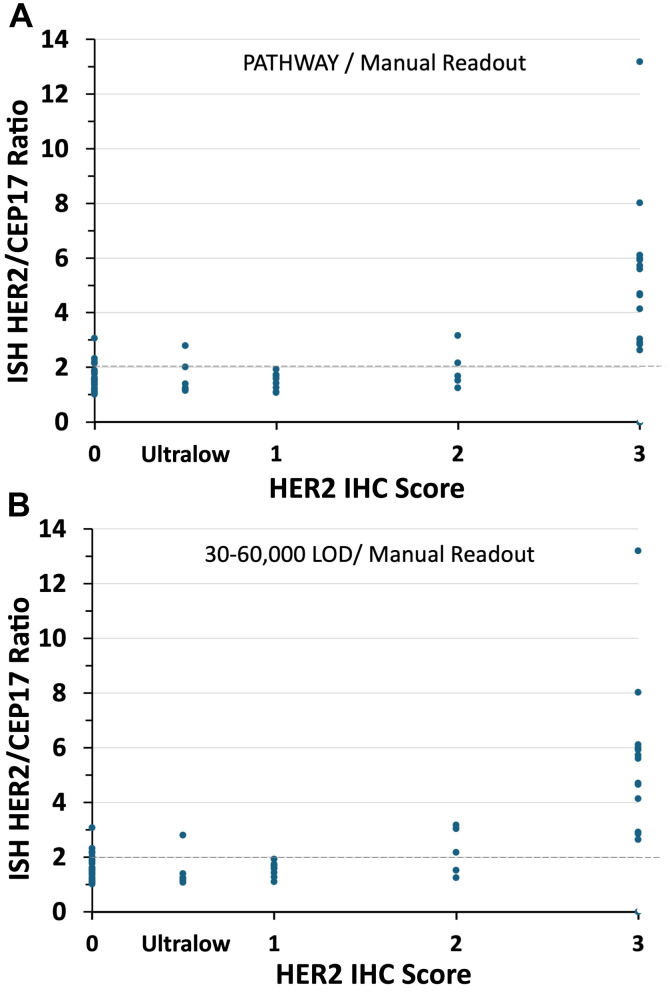
HER2 gene amplification, as measured by *in situ* hybridisation (ISH score, y axis), as a function of the consensus HER2 score from participating laboratories using the Roche PATHWAY HER2 assay (panel A), or all assays with an LOD between 30,000 and 60,000 molecules/cell equivalent (panel B). Other assays that do not fit these criteria are not shown. The dots represent data from tissue cores in the tissue microarray, from labs that fit the criteria cited at the top of each graph.

Since the DB04 clinical trial assay closely aligned with the “4B5 per IFU” group, this study primarily focused on that assay. As an initial step, we evaluated the suitability of the two 4B5 groups (“per IFU” and “LDT”) in accurately assigning patients to Trastuzumab treatment. The Roche 4B5 PATHWAY HER2 assay (“4B5 per IFU” group) distinguished between HER2 gene-amplified from unamplified tissue samples (Fig. 3A). Each dot in Fig. 3A represents the HER2/CEP17 *in situ* hybridisation (ISH) score (y axis) for a TMA tissue core. Each tissue sample's HER2 score (x axis) is the consensus of labs in the “4B5 per IFU” group. Fig. 3A illustrates that HER2 3+ scores are strongly associated with gene-amplification. The non-amplified samples mainly fell into the 0–2+ range (Fig. 3A). Nonetheless, six samples with HER2 scores less than 3+, out of a total of 70 that could be evaluated by ISH (8.6%), had HER2/CEP17 ratios between 2 and 3.2.

To calculate diagnostic sensitivity and specificity, each TMA core was classified as TP, TP, TP, or false negative based on the most recent ASCO/CAP HER2 scoring guidelines,^22^ based on the HER2/CEP17 ratio. In this classification, a 2+ HER2 score was either a TP or TP depending on the ISH data. Seven cases (out of 80) in the TMA could not be analysed by ISH for technical reasons. Using ISH dual probe scores as a gold standard assay, diagnostic sensitivity of the “4B5 per IFU” assay is 85.7% (18/21, 95% confidence limits (CL) 63.66–96.95%), specificity 100% (49/49, 95% CL 92.75–100%), positive predictive value 100% (18/18, 95% CL 81.47–100%), and negative predictive value 94.2% (49/52, 95% CL 84.05–98.79%).

The sensitivity measurement is likely an under-estimate. Sensitivity and negative predictive value parameters were impacted by three false negative cases that showed HER2 gene amplification by ISH but HER2 scores less than 2+. These patients would not have been offered Trastuzumab because ISH would not typically have been performed with a HER2 score below 2+. It is possible that the protein but not DNA degraded during extended storage, as the tissue blocks were often more than ten years old. It is also possible that these cases showed HER2 gene amplification that did not translate to an increased HER2 protein concentration. Finally, it is also possible that these cases are TP for HER2 gene amplification.

Despite these few exceptions, the “4B5 per IFU” assay performed exceptionally well for stratifying patients to Trastuzumab therapy when ISH is used as a gold standard assay. The assay accurately identified tumours with high HER2 concentrations associated with a 3+ score even though it had the lowest analytic sensitivity among the various commercial assays shown in Fig. 2. Therefore, high analytic sensitivity is not essential for accurately identifying patients for Trastuzumab therapy. To further define the group in terms of LOD, we graphed the data as a function of all labs having an analytic sensitivity in the 30,000–60,000 range (Fig. 3B). This includes not only the PATHWAY HER2 assay but also numerous laboratory-developed assays using the same 4B5 primary antibody as well as other commercially available assays. The data for all assays with LODs between 30 and 60,000 molecules/cell equivalent, the range that matches the PATHWAY assay, is essentially identical (Fig. 3B).

### HER2 assay dynamic ranges

Since there is no ISH-equivalent gold standard assay for HER2-low scores, we applied a new concept for evaluating the suitability of HER2 IHC assays for HER2-low scoring. We assessed HER2 assays' dynamic range, as described in Methods/Assessment of HER2 IHC Dynamic Range and Fig. 1. This assessment relies on the expectation that increasing analytic sensitivity (lower LODs) should translate to higher average HER2 scores. Average HER2 scores are calculated from the consensus scores of all the TMA cores, for a particular readout method, and at a specified LOD (Methods). LOD is calculated from calibrator data (Methods). If an assay has a dynamic range, then there will be higher average HER2 scores as analytic sensitivity increases (lower LODs). The absence of a dynamic range means that the scores do not correlate with HER2 expression.

**Fig. 4:**
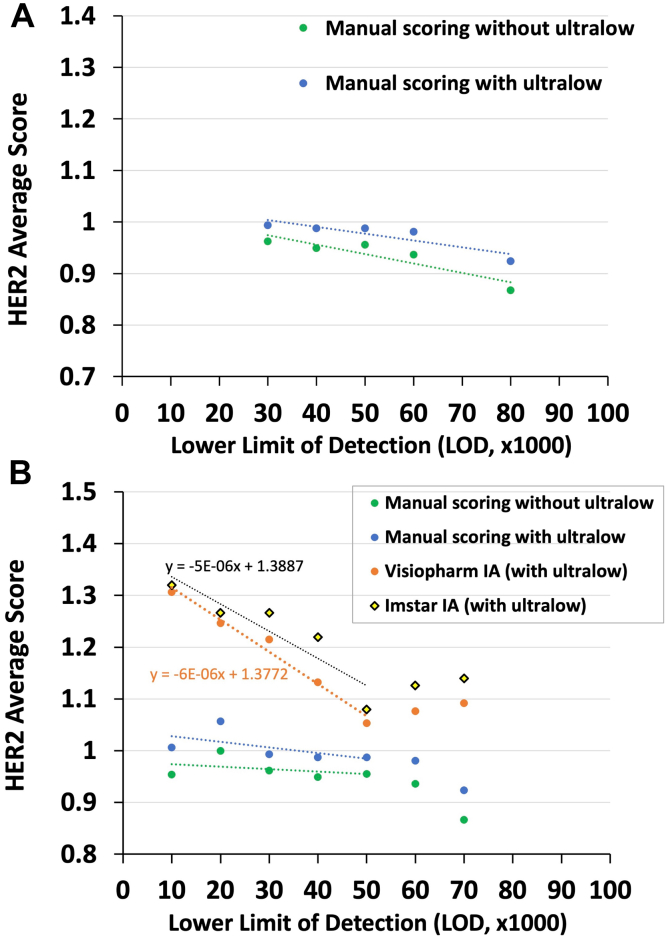
Average HER2 score of the tissue cores (y axis) as a function of assay analytic sensitivity (LOD, x axis). Participating laboratories were grouped according to their HER2 assay LOD ± 5000, as indicated on the x axis. Panel A shows data from laboratories using the PATHWAY assay, scored manually, and with (blue line) or without (green line) the ultralow category. There is little change in the average HER2 scores with LODs from 30,000 to 60,000. Panel B illustrates the effect of incorporating both image analysis and more sensitive HER2 IHC assays, extending both lines to the left on the x axis. The x axis of each is intended to be multiplied by 1000.

Fig. 4A expresses the relationship between average HER2 scores (y axis) as a function of measured analytic sensitivity (LOD, x axis), for the Roche PATHWAY assay. Fig. 4A reveals that the correlation is weak or absent. The blue and green linear regression lines (Fig. 4A) have a shallow slope due to a single data point—the average HER2 score at the 80,000 LOD. For LODs of 30,000–60,000, using the standard ASCO/CAP scoring criteria (without the ultralow scoring category), there is almost no change in the average HER2 score (green data points). Within the 30,000–60,000 LOD range, there is no linear relationship between the average HER2 score and LOD, i.e., the slope is not significantly different from zero (p = 0.195). For this initial analysis (green dashed line), ultralow scores were considered HER2 0 since that is how they are treated per the ASCO/CAP scoring criteria. Amending the ASCO/CAP guidelines by inserting an ultralow category (blue dashed line) does not make much difference. It only creates a small, statistically significant (p = 0.02) constant positive bias relative to the data points without an ultralow scoring category (Fig. 4A, comparing the blue to the green data points) but the slope is essentially unchanged (Fig. 4A, blue line, p = 0.725). As before, there is no linear relationship between the average HER2 score (including the ultralow category) and LOD (p = 0.395). Therefore, HER2 IHC assays of the same commercial type as used in the DB04 trial—with or without the ultralow score category—are not well suited for finely distinguishing low positive scores.

We then evaluated two additional interventions for their effect on assay dynamic range: (a) evaluating more sensitive HER2 assays and (b) HER2 scoring using image analysis (Fig. 4B). Some HER2 IHC assays have higher analytic sensitivities than PATHWAY (Fig. 2). Fig. 4B includes these HER2 assays with lower LODs. For Fig. 4B, the slopes and calculations are taken from data points below 50,000 LOD because the more sensitive HER2 assays with image analysis: (a) have LODs below that point, and (b) lead to a visual breakpoint in the data (orange line).

Incorporating more sensitive assays did not materially change the slopes of the two lines (Fig. 4B, green and blue dashed lines) when using manual scoring. For data points below 50,000 LOD, there was no significant linear relationship between the average HER2 score (y axis, Fig. 4B) and LOD, regardless of whether the ultralow category is included (p = 0.69) or not (p = 0.51).

This may be due to differences in the readout methods. While LOD calculations are performed on calibrators with their own dedicated image analysis readout method, tissue samples are read by pathologists. The resulting analytic sensitivity (LOD) from calibrators reflects the assay's technical capability but does not account for interpretive inconsistency at the readout stage—particularly in the ultralow and HER2-low ranges, where inter-observer variability is high.

Image analysis of tissue staining was then evaluated to investigate if it can improve the assay dynamic range when it replaces manual readouts. There is a six-fold increase in the slope when Visiopharm image analysis is coupled with the more sensitive assays, which is significantly different than manual readouts (p = 0.0017, Fig. 4B, orange line). Similar data are seen with image analysis using the ImstarDx software (Fig. 4B, yellow diamonds). From the exact same stained slides as used in pathologist readouts, image analysis discerns increases in staining proportional to increasing analytic sensitivity (Fig. 4B, orange dashed and black dotted lines). This type of validation for image analysis is new, as it is independent of the pathologist score. Currently, the pathologist score is considered the gold standard against which image analysis was judged.

**Fig. 5:**
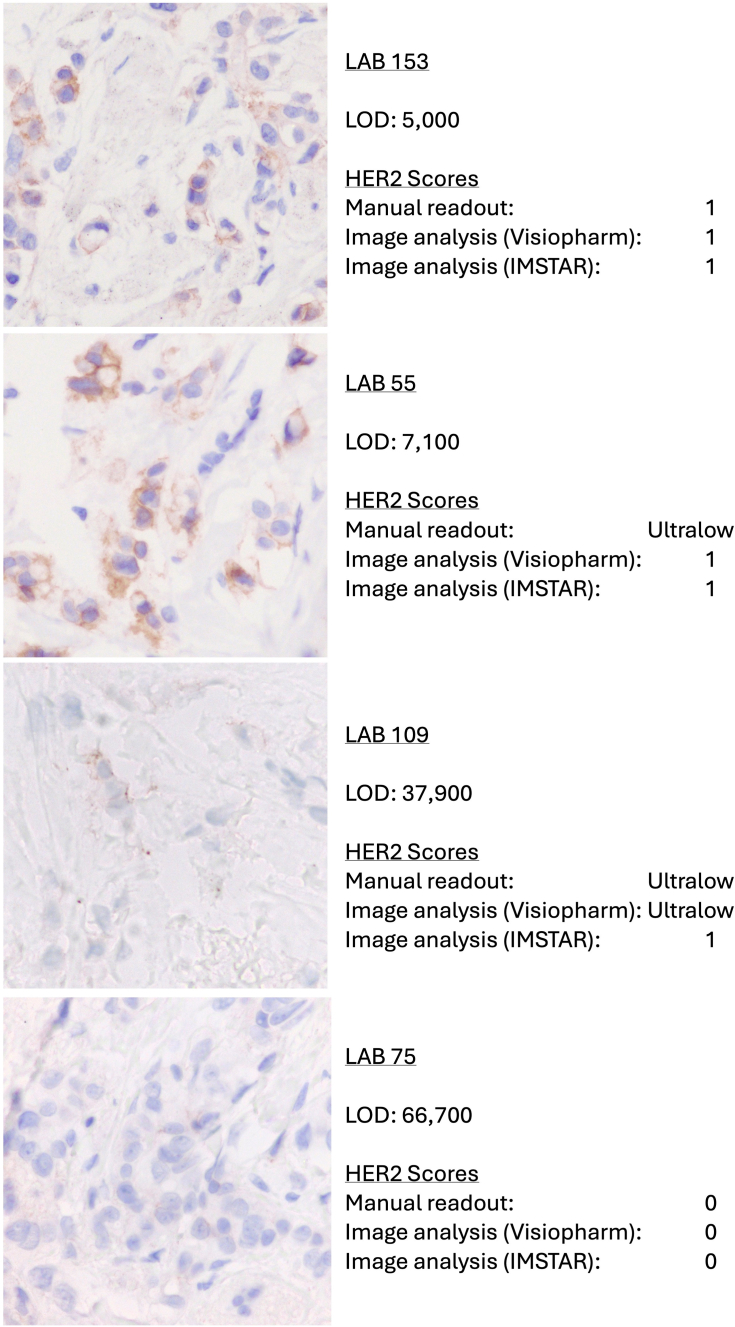
Photomicrographs of serial sections of tissue core B13 in the TMA, from four IHC laboratories. The LOD and HER2 scores are as indicated on the right. Magnification 400×.

Fig. 5 shows exemplary photomicrographs from which the data in Fig. 4 are derived, for four IHC laboratories. Each of the images are from tissue core B13 in the TMA. The images show dramatically improved staining at the HER2-low end of the scale using the highly sensitive mHercepTest assay (Labs 153 and 55). Assays with higher LODs, as illustrated for Labs 109 and 75, are insufficiently sensitive to detect HER2 in these cases.

### The HER2 0 score dynamic range is more responsive

**Fig. 6:**
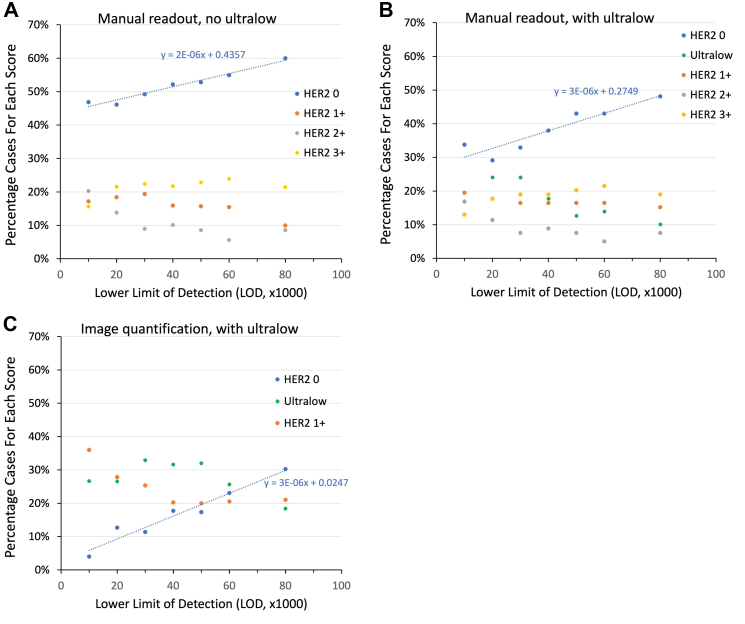
Correlation of individual HER2 scores as a function of assay analytic sensitivity (LOD), as scored manually without the ultralow category (panel A), manually with the ultralow category (panel B), and using Visiopharm image analysis with the ultralow category (panel C). The HER2 0 category of scores includes a blue regression line, showing a positive correlation in all three conditions. For panel C, the HER2 2+ and 3+ categories are not shown to better illustrate the changes with the HER2 0, ultralow, and 1+ data.

Fig. 6 dissects out the individual scores from the average HER2 score data of Fig. 4. It illustrates more granular detail from Fig. 4, showing the percentages of cases with each HER2 score, at each LOD. Fig. 6 illustrates the effect of assay analytic sensitivity (x axis) on the percentage of cases for each HER2 score (y axis) from: manual scoring without the ultralow category (Fig. 6A), manual scoring with the ultralow category (Fig. 6B), and image analysis with the ultralow category (Fig. 6C). Unlike Fig. 4, the data in Fig. 6 are percentages of cases in the TMA for each HER2 score (y axis). These individual score percentages collectively comprise the average scores of Fig. 4.

Fig. 6A–C shows a direct relationship between analytic sensitivity (LOD, x axis) and the HER2 0 score (blue linear regression line). Increasing analytic sensitivity (lower LOD scores) results in a declining percentage of patients with HER2 0 test scores. Such a relationship makes sense. With increasing analytic sensitivity, some HER2 0 cases are recognised as ultralow or 1+. Therefore, even though the aggregate average HER2 scores did not correlate with analytic sensitivity (Fig. 4A and B) when assessed manually, the HER2 0 score does. The HER2 0 correlations with LOD are statistically significant (p = 0.0001, Fig. 6A; p = 0.0032, Fig. 6B; p = 0.00041, Fig. 6C). This suggests that while the average HER2 score demonstrated a poor dynamic range (Fig. 4), pathologists are often able to visually distinguish no staining (HER2 0) from some staining. This finding suggests that accuracy of HER2-low scores using highly sensitive HER2 assays is limited by inter-observer variability. Image analysis can address this limitation., allowing a fine distinction among HER-2 low scores. Even without image analysis, pathologists are somewhat able to identify HER2 0 from non-zero, with increasing numbers of HER2 0 as less sensitive HER2 assays are used.

### Clinical trial assay sensitivity impact modelling

The reported challenges in reliably reproducing the DB04 clinical trial assay in actual practice^23^ highlight the importance of assay standardisation. Defining the analytic sensitivity of a clinical trial assay is an important component of assay standardisation for subsequent clinical implementation. While this may seem intuitively clear, no studies to our knowledge have demonstrated the actual consequences of misalignment between the clinical trial assay and the assay as implemented in clinical practice. Rather than quantitatively measuring LOD, analytic sensitivity in both settings is typically inferred through tissue sample staining, despite unknown levels of biomarker expression in these samples. The CASI-01 study data provide a valuable opportunity to illustrate the patient care consequences of not measuring analytic sensitivity of a companion diagnostic test in a clinical trial. Using calibrators to define the critical LOD of IHC assays used in clinical trials might facilitate the transfer of IHC methods from these trials to diagnostic use.

We analysed the CASI-01 data, modelling the impact of an analytic sensitivity mismatch between the clinical trial assay and commercial assay used in clinical IHC laboratories. Since the analytic sensitivity of neither is measured, the consequences of a mismatch are unknown. If the clinical trial assay is highly sensitive and the commercial version is less sensitive, or vice versa, what are the patient care consequences? The CASI-01 study data allow for a direct demonstration to be modelled.

**Fig. 7:**
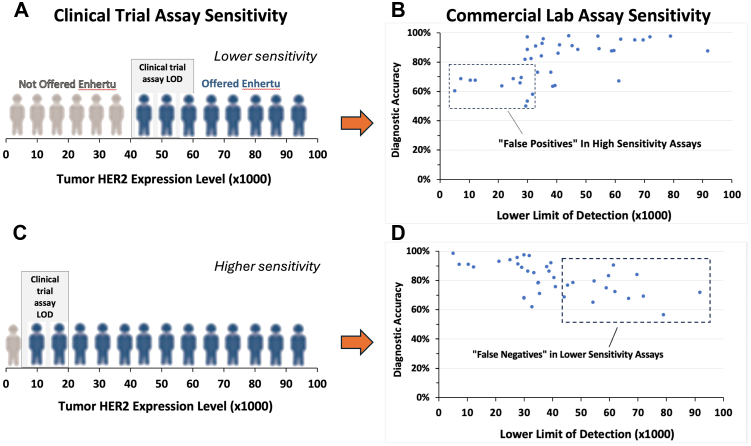
Modelling the effect of analytic sensitivity on diagnostic accuracy. These graphs simulate (with CASI-01 data) the consequences of two contrasting HER2 clinical trial assays: (1) a clinical trial assay that is relatively insensitive, with an LOD >40,000 (A), or (2) the clinical trial assay is highly sensitive, with an LOD in the <20,000 (C). Only patients with HER2 tumour cell concentrations higher than the LOD will show staining and therefore receive ADC treatment (blue persons, A and C). When those criteria are applied to selecting the correct patients for treatment, then diagnostic accuracy of the participating IHC laboratories is depicted in panels B & D. For these graphs (B and D), the TMA scoring data incorporate the ultralow category and are based on image analysis.

Fig. 7 demonstrates the impact of two distinct clinical trial assays with different limits of detection (LODs), >40,000 and <20,000, on diagnostic accuracy and the correct assignment of patients to T-Dxd treatment. Assays with these LODs are represented among the various labs in the survey. Fig. 7A schematically illustrates a clinical trial HER2 IHC assay with an LOD >40,000 (x-axis), representing a less sensitive test. In this scenario, patients whose carcinomas express HER2 levels above 40,000 are classified as HER2-low positive and are potential candidates for T-Dxd (indicated as blue figures in Fig. 7A). For these patients, the decision to administer T-Dxd is considered the correct outcome and is compared to other labs' IHC results. If the test results from other HER2 assays, each with their own LOD, align with this patient treatment decision for T-Dxd, then they are also considered TP or TN. Conversely, if the treatment decision differs, the results are classified as FP or FN.

Using the TP, TN, FP, and FN designations, we calculated diagnostic accuracy for each laboratory and plotted the data as a function of LOD (Fig. 7B). Each dot in the graph represents the diagnostic accuracy of a distinct laboratory. Diagnostic accuracy measures a laboratory's ability to assign patients to T-Dxd treatment in alignment with the consensus among labs with an LOD >40,000. Labs with LODs ≥40,000 exhibit the highest diagnostic accuracy (Fig. 7B, y-axis). More sensitive assays with lower LODs display a 20–30% reduction in accuracy due to TP staining of tumours with lower HER2 concentrations (Fig. 7B).

In a second contrasting scenario, the clinical trial assay is highly sensitive, with an LOD of <20,000 (Fig. 6C). More patients are assigned to treatment (blue people of Fig. 7C) than the lower sensitivity assay (Fig. 7A). The impact on diagnostic accuracy is illustrated in Fig. 7D. In this second analysis, IHC labs with LODs <20,000 are used in determining the correct treatment decision. Diagnostic accuracy is then measured for all the labs. As expected, the highest sensitivity labs show the highest diagnostic accuracy. They are the ones correctly assigning patients to ADC treatment. Fig. 7D shows the extent of the decline (30–40%) when using HER2 IHC assays with lower analytic sensitivity (higher LODs). These data (Fig. 7B and D) illustrate the importance of: (1) selecting an analytic sensitivity that accurately distinguishes responders from non-responders, and (2) once that is accomplished, aligning the analytic sensitivity of IHC assays in clinical practice with the original clinical trial assay used for identifying patients who will benefit from treatment.

## Discussion

The principal goal of this study is to generate data-driven guidance to clinical laboratories trying to perform accurate HER2 testing. We sought to identify the analytic sensitivity (LOD) cutoffs associated with accurate patient stratification for Trastuzumab and T-Dxd treatment. Since IHC calibration is new, no such analysis was performed as part of regulatory clearance or afterwards.

The data show that currently used HER2 IHC assay LODs are fit for purpose for identifying tumours with amplified HER2 gene and overexpressed HER2 (3+) protein (Fig. 2). Nonetheless, there was a surprising amount of variability in analytic sensitivity among IHC laboratories using an FDA-cleared HER2 kit. Among the 18 surveyed IHC labs using the unmodified Roche PATHWAY HER2 test, the mean LOD was 54,436 ± 19,569, representing a 36% interlaboratory coefficient of variation (CV). The fact that they accurately assigned HER2 gene-amplified cases to the 3+ score (Fig. 2) suggests that a 3+ score likely corresponds to a much higher level of HER2 expression. As a result, highly sensitive assays are not required to accurately identify HER2 3+ tumours. LODs in the 10,000–60,000 range, as is common for many HER2 assays, are perfectly suitable.

An additional study goal was to determine if an LOD cutoff could be identified that would better distinguish gene-amplified HER2 2+ from non-amplified HER2 2+. This might decrease the need for reflex ISH testing. Since HER2 gene amplification correlates with HER2 protein expression,^24^ there should be a protein expression level that helps shrink the 2+ grey zone, which requires ISH testing. We could not identify such a cutoff. A possible underlying reason is that the tissue blocks were old, often more than ten years, per Institutional Review Board requirements. Consequently, HER2 protein levels may have degraded. Some tissue sections that now stain as 2+ may have been 3+ at the time of original diagnosis.

HER2-low is a different story. Testing fitness for purpose of HER2-low readout required a different experimental design because there is no gold standard assay for this range of HER2 staining. Therapeutic response, such as in a clinical study, may be the desired clinical purpose but it is not an assay gold standard for biomarker expression levels. In the absence of a HER2-low gold standard, we evaluated a new parameter - IHC dynamic range. However, no method exists for its measurement. Therefore, we developed a new method—an evaluation whether the HER2-low scores of ultralow, 1+, and 2+ are influenced by the HER2 analytic sensitivity. More sensitive assays should produce more positive results (Fig. 1). CASI-01 participating laboratories represent a broad range of analytic sensitivities, each of which tested 80 diverse breast carcinomas in a TMA. The dynamic range evaluation examined the aggregate HER2-low scores vs. analytic sensitivity relationship, a surrogate for a traditional dynamic range evaluation.

The data demonstrate that the assay conditions and readout method used for the PATHWAY assay, the same as used in the DB04 and DB06 trials, are ineffective in distinguishing HER2-low levels of expression. There was little to no dynamic range at the HER2-low end (Fig. 4). The insufficient dynamic range can be overcome by using more sensitive tests and digital image analysis. High analytic sensitivity both extends the range of detection and increases the stain intensity, rendering weak stains easier to appreciate visually (Fig. 5). Objective image analysis algorithms offer improved readout accuracy and reproducibility. Our data show that the combination of higher assay sensitivity and the precision of image analysis leads to superior assay dynamic range, as predicted by Fig. 1.

When the individual HER2 scores were teased out, the percentage of HER2 0 cases correlated with LOD (Fig. 6A and B, blue line). As expected, higher sensitivity (lower LODs) led to lower percentages of HER2 0, even with manual readouts (Fig. 6A and B). This finding suggests that detecting no staining at all (HER2 0) is more amenable to reproducible visual interpretation than distinguishing ultralow, 1+, and 2+. This correlation is important because it is the entry criterion for T-Dxd treatment.

The improvement in assay dynamic range that is achieved with image analysis (Fig. 4B) underscores the critical role of the HER2 readout. Normally, the pathologist readout is taken as the gold standard when evaluating image analysis algorithms. In this instance, the image analysis data demonstrate a superior correlation with analytic sensitivity for low HER2 expression levels (Fig. 4B). The fact that image analysis tracks so well with analytic sensitivity (LOD) emphasises the limitations of visual readouts in certain diagnostic contexts. Both the Visiopharm and Imstar image analysis algorithms use a mathematical representation of the ASCO/CAP guidelines in performing HER2 scoring. It seems reasonable to conclude that the image analysis algorithms are better able to distinguish faint staining gradations that more accurately track with analytic sensitivity.

While our findings underscore the advantages of image analysis in expanding the dynamic range and improving reproducibility—especially in the challenging HER2-low expression range—we also recognise that these benefits depend on the quality and consistency of input images. For this study, the Visiopharm analyses were based on images from a single scanner (Roche DP-200). Similarly, the Imstar Dx analyses were similarly based on a single scanner (Pathfinder automated scanner composed of a BX63 microscope and a Märshäuzer slide-loader). For clinical laboratories using image analysis algorithms for HER2 classification support, the imaging protocols and scanner specifications are important for ensuring reliable algorithm. The performance of image analysis in routine clinical practice can be influenced by several technical factors, including image resolution, autofocus accuracy, Z-stacking (depth of field), standardisation of tumour reproduction, and file compression. These variables can affect the consistency of algorithm outputs and merit careful quality control and standardisation, especially if the images are to be used for routine clinical diagnostic purposes.

A limitation of our study is that the data do not identify an optimal LOD cutoff for T-Dxd treatment. Only a clinical trial can clinically validate biomarker assays for a cutoff. While higher analytic sensitivity and image analysis extend the HER2 assay dynamic range, the assay conditions are not directly translatable to the already-established Trastuzumab or T-Dxd treatment criteria. The optimal LOD cutoff would need to be established under any new assay condition.

The use of more sensitive HER2 assays could theoretically shift HER2 scores upwards, i.e., changing from HER2 0 to 1+, or from 1+ to 2+, etc. While this may improve detection of patients eligible for treatment with T-Dxd, it may potentially also lead to inappropriate treatment stratification for Trastuzumab. This underscores the importance of using IHC assays with known analytic sensitivity during clinical trials, and then matching those assays in clinical practice. To avoid patient treatment misclassification, one potential approach is to reserve the high sensitivity HER2 assays for patients for T-Dxd while less sensitive assays are used for determining Trastuzumab eligibility. This “fit for purpose” testing scheme might avoid the potential hazard of shifting HER2 2+/ISH− to HER2 3+ results.

Another limitation of this study is that it was not designed to evaluate reader variability in HER2 scoring. There are already excellent published studies on that topic.^25^, ^26^, ^27^ The manual HER2 readouts were performed by four experienced breast pathologists, with each TMA being scored by one. This most closely replicates professional practice and was also necessary logistically. With 54 participating labs, each staining a TMA with 80 samples, there were over 4000 TMA cores to analyse. It is possible that the use of consensus scores from multiple pathologists would lead to more accurate HER2 manual scoring and a better HER2-low dynamic range. However, it's not reflective of real-world surgical pathology practice. It's also not scalable from a clinical trial to clinical practice because it's an acknowledgement of an inherently subjective evaluation. To scale a clinical trial assay to worldwide implementation, the readout needs to be reproducible. Either the signal to noise ratio of the readout is large enough for reproducible visual (pathologist) interpretation or a validated IA algorithm performs the readout.

Another limitation of this study is that TMA cores can potentially vary through the depth of the paraffin block. This potential source of variability is in addition to the variability among laboratories and readers. With all these sources of variability, and among 80 different cores, we could not discern any patterns relating to the plane of section in the paraffin block.

The data were also analysed to illustrate the impact of mismatches between the clinical trial assay and the commercial assay. There are no regulatory requirements to measure analytic sensitivity. Therefore, it is unknown if and to what extent such mismatches arise. It is also unknown whether drift in analytic sensitivity occurs over time in a commercial IHC assay. Fig. 7 illustrates the impact of such mismatches or drift in analytic sensitivity in the context of HER2 testing for T-Dxd eligibility. The data reinforce the need for measuring analytic sensitivity on a clinical trial assay, enabling better methodology transfer for routine clinical laboratory use.

Despite the fact that our study does not include patient treatment response data, there are three clinical laboratory recommendations that are reasonably well supported:

1. For HER2 testing in the context of Trastuzumab, analytic sensitivity appears less critical. An LOD as high as 60,000 molecules/cell equivalent (microbead) will detect HER2 3+ cases.
2. For HER2 testing in the context of T-Dxd testing using the PATHWAY HER2 IHC assay, analytic sensitivity should ideally be closer to the analytic sensitivity plateau for that method, i.e., 30,000–40,000 molecules/cell equivalent. While the DB04 and 06 clinical trial assay analytic sensitivities are unknown, it is likely that the assays were appropriately optimised, achieving the analytic sensitivity listed above. For labs using the PATHWAY assay, it seems reasonable to try to replicate the 30,000–40,000 analytic sensitivity.
3. Greater analytic sensitivity, such as with the monoclonal HercepTest, will identify more HER2 ultralow and 1+ cases. At a minimum, a negative staining result with higher sensitivity will more reliably identify a TP, i.e., a greater negative predictive value. However, clinical outcomes data using such highly sensitive HER2 assays are lacking (to our knowledge), so the optimal treatment cutoff is unknown.

To summarise, the study demonstrates that HER2 IHC LODs ≤60,000 molecules/cell equivalent are readily achievable and accurately identify HER2 overexpressing (3+ and 2+ amplified). The advent of T-Dxd, on the other hand, has upset the status quo of HER2 scoring, stretching a long-established diagnostic IHC algorithm beyond its current capability.^23^ The solution, we believe, is to treat IHC as an assay rather than a stain, incorporating the types of quality assurance and measurement techniques that represent a standard of care for immunoassays.^28^, ^29^, ^30^ This includes identifying assay dynamic range and locking in quantitative analytic assay requirements when demonstrating fitness for purpose. Only in this way will a favourable clinical trial result be accurately translated for application to cancer patients across the globe.

## Contributors

D.J.D., J.Y., C.S., B.C. were responsible for assigning HER2 scores to TMA samples; H.H., J.T., F.S., A.P., and M.C. performed image analysis on TMA samples; S.R.S. analysed stained calibrators and calculated LOD; S.B. analysed the data, provided central coordination, and drafted the manuscript; S.P.N. selected tissue cores for inclusion into the TMA; K.V. prepared the calibrators; S.N., S.C.P., J.S., H.H., J.T., D.J.D., E.T., K.M., L.C., M.K., M.J.S., B.J., N.H. contributed to the experimental design, reviewed and critiqued the manuscript; Y.Y. performed *in situ* hybridisation on TMA samples; S.N., S.C.P. provided operational support by coordinating the participation of IHC laboratories through their proficiency testing network; J.S. provided statistical support. All authors read and approved the final paper. S.B. and J.S. accessed and verified the underlying data.

## Data sharing statement

The data that support the findings of this study are available from the corresponding author upon reasonable request.

## Declaration of interests

SRS, KV, and SB are officers and stockholders in Boston Cell Standards; HH and JT are employees in Visiopharm A/S; MC, FS, and AP are employees of ImstarDx; MK serves on an advisory Board to AstraZeneca, ET discloses educational grants from Roche and Eli Lilly, consulting with Boehringer Ingelheim and Agilent, and leadership roles in IQN Path and CBQA. DJD, SN, SCP, JY, CS, BC, SPN, YY, JS, NH, MJS, BJ, LC, and KM declare no competing interests relevant to this paper.

## Appendix A Supplementary data

Captions for Supplementary Figs. S1–S6
