# Supplementary material for: New standards in HER2-low testing: the CASI-01 comparative methods study
Source: eBioMedicine. 2025 Sep 12;120:105919. doi: 10.1016/j.ebiom.2025.105919 (PMC12571579; doi:10.1016/j.ebiom.2025.105919)
Supplement: Captions for Supplementary Figs. S1–S6 [file mmc1.docx]

Supplementary Figure 1. Photomicrograph of the stained TMA, in four rows of 20. Three irrelevant orientation cores are in the lower left. Often, several cores detach during processing, as shown, and are not included in the analysis. The inset explains the numbering system for tracking and reporting the scores.

Supplementary Figure 2. Age distribution of patients from whom the tumour cores were derived, for incorporation into a TMA.

Supplementary Figure 3. Lymph node status of patients from whom the tumour cores were derived, for incorporation into a TMA. pNX: Lymph node status could not be assessed. pN0 or yPN0: No regional lymph node metastasis identified or isolate tumour cells only. ypN0: No positive lymph nodes. pN1a: Metastases in 1–3 axillary lymph nodes, at least one metastasis >2.0 mm. pN1mi: Micrometastases (~200 cells, >0.2 mm, but ≤2.0 mm). pN2: Metastases in 4–9 axillary lymph nodes; or positive ipsilateral internal mammary lymph nodes by imaging in the absence of axillary lymph node metastases. ypN2a: 4–9 positive lymph nodes.

Supplementary Figure 4. Tumour grade of patients from whom the tumour cores were derived, for incorporation into a TMA.

Supplementary Figure 5. IHC calibrator spots on a microscope slide, in a 3 x 5 array. The numbers 1, 5, 6, and 10 refer to the orientation of calibrator “levels”, with level 1 being the lowest and 10 the highest. The concentrations for each level are specified in a Certificate of Analysis. The middle row is of negative controls. Each spot is comprised of approximately 5000 microbeads coated with HER2 peptide. The inset shows an example of stained cell-sized microbeads as seen under microscopic examination.

Supplementary Figure 6. Logic table for converting test results from participating laboratories (“Observed result”, y axis) to TP, TN, FP, and FN. This table is applied in the context of HER2-low scoring for T-Dxd. The designation “0.5” refers to an ultralow result. The designation “U” means unscored due to technical limitations such as loss of tissue. The combination of 3+ (gold standard) and 2+ (observed result) is designated TP because the sample would be tested by ISH and the patient treated appropriately. The combination of 1+ (gold standard) and 3+ (observed result) was denoted “TP” but defies classification because patients would be treated with Trastuzumab instead of T-Dxd. There were no instances like this.
